# Research Metrics for Health Science Schools: A Conceptual Exploration and Proposal

**DOI:** 10.3389/frma.2022.817821

**Published:** 2022-04-25

**Authors:** Nigussie Gemechu, Meghan Werbick, Michelle Yang, Adnan A. Hyder

**Affiliations:** ^1^Milken Institute School of Public Health, George Washington University, Washington, DC, United States; ^2^Center on Commercial Determinants of Health, Milken Institute School of Public Health, George Washington University, Washington, DC, United States; ^3^Department of Global Health, Center on Commercial Determinants of Health, Milken Institute School of Public Health, George Washington University, Washington, DC, United States

**Keywords:** health research, research metrics, public health, research outcomes, research impact, research measurement

## Abstract

Research is a critical component of the public health enterprise, and a key component of universities and schools of public health and medicine. To satisfy varying levels of stakeholders in the field of public health research, accurately measuring the return on investment (ROI) is important; unfortunately, there is no approach or set of defined metrics that are universally accepted for such assessment. We propose a research metrics framework to address this gap in higher education. After a selected review of existing frameworks, we identified seven elements of the generic research lifecycle (five internal to an institution and two external). A systems approach was then used to broadly define four parts of each element: inputs, processes, outputs, and outcomes (or impacts). Inputs include variables necessary to execute research activities such as human capital and finances. Processes are the pathways of measurement to track research performance through all phases of a study. Outputs entail immediate products from research; and outcomes/impacts demonstrate the contribution research makes within and beyond an institution. This framework enables the tracking and measurement of research investments to outcomes. We acknowledge some of the challenges in applying this framework including the lack of standardization in research metrics, disagreement on defining impact among stakeholders, and limitations in resources for implementing the framework and collecting relevant data. However, we suggest that this proposed framework is a systematic way to raise awareness about the role of research and standardize the measurement of ROI across health science schools and universities.

Accurately measuring the return on investment (ROI) is a critical yet often overlooked tenant of public health research and practice. Despite its ability to inform future investments, maximize benefits, and improve interventions, the field of public health lacks a standardized framework to measure such returns. In this paper, we propose a framework that can help higher educational research institutions measure the performance of various research projects. We hope that by utilizing a common framework, health science schools and universities can maximize the value of their research and their impact on the field of public health.

## Introduction

Public health research contributes immensely to scientific breakthroughs, healthier communities, innovations, sound policies, and economic growth (Gostin et al., 2009). Public health research has a diverse set of stakeholders at varying levels including funders, policymakers, researchers, participants, and members of the community; and many of these stakeholders have an interest in quantifying the return on investment (ROI) for research (Cruz Rivera et al., 2017). However, despite some existing models, there is no universally accepted set of metrics to assess ROI for public health research; despite a growing demand to identify and develop standards to effectively measure and communicate research-related progress and success (Banzi et al., 2011). In addition, challenges such as inconsistent definitions of outcomes, time lapse between research and impact, issues in establishing attribution, and accommodating stakeholder perceptions make this goal complex (Graham et al., 2018). A universally accepted approach however, would provide a system to measure social, economic, and environmental benefits of research.

Research systems exist at multiple levels—institutional, state, national, and international—and some approaches to measuring them have been published (Hanney et al., 2020). Research measurement systems need to evolve and strengthen by including new dimensions and measures for each level (David and Joseph, 2014). Academic institutions often have their own systems tracking selected metrics within the context of their research ecosystem needs; however, they are often *ad-hoc*, limited in scope and informal in nature (Aguinis et al., 2020). Institutionalizing consistent and universal standards, such as definitions, data collection, analysis and reporting, would help improve practices in research tracking and measurement. In addition, having clear research strategic plans and goals would also optimize the selection and implementation of appropriate research metrics (Hanney et al., 2020).

Useful metrics provide valuable insights on the research process and enables benchmarking based on standard definitions for meaningful comparisons (University of Birmingham, 2021). Building a research measurement system and tools can enable academic institutions collect and provide research metrics on a consistent and reliable basis; and enables multidimensional ways of assessing the value and ROI of research. And an effective impact assessment framework can demonstrate how positive change resulting from research investments improves prioritization, decision-making, management of stakeholder expectations and promotes accountability and transparency at an institution.

The overall goal of this paper is to explore research measurement frameworks that are suitable for an academic school level application. Our objective was to propose a research measurement framework and explore its applicability in an academic setting in the United States.

The proposed framework focuses on research metrics within an academic institutional research ecosystem; to help guide research assessment and planning. We hope that this paper will lead to further application and testing of our proposed approach in other settings in the US and globally.

## Selected Review of Existing Frameworks

A number of research assessment frameworks have been developed and implemented in specific institutions or countries, over the past years (Guthrie et al., 2013). However, there are no guidelines or consensus either around one universal framework or even around the protocol to develop a methodological framework; hence reviewing some commonly used approaches was essential (Cruz Rivera et al., 2017). Therefore, we reviewed frameworks that were implemented at different scale to address research metrics and measurement issues (Table 1). All reviewed frameworks have been applied in specific setting or location; are not universally acclaimed; and none have been recommended for academic institutions. In addition, many suffer from other limitations such as cost, complexity, data collection burden, underdevelopment of definitions, and issues of scale (Table 1).

**Table 1:** Descriptions of selected research assessment frameworks.

**Framework**	**Description/ Purpose**	**Scope**	**Methods/ Data Sources**	**Advantages**	**Limitations**
Canadian Academy of Health Science (CAHS)—Formerly Payback Framework	A framework developed using a logic model for health research translation, and drawing on a library of indicators. It aims to provide consistency and comparability between institutions in a research system with multiple regional funders. Provides a framework for consistent data gathering and presentation across a series of case studies	Five categories: advancing knowledge; capacity building; informing policies and product development; health and health sector benefits; broader economic benefits; categories cover range of perspectives that important to both researchers and various types of users	Review of documents/archives, surveys, analysis of publications, interviews, Bibliometrics, scoring	Tailored to Canadian context; very comprehensive; flexible—applies to range of types of funding, and different types of research; developed through engagement; formative; looks at process, outputs and impacts; aligned with main funders in Canada	Resource intensive to implement; complicated; developed by committee; ambiguity in definitions between outputs and outcomes; impose burden; no ranking; approach plays down difficulties of attribution to specific studies
Excellence in Research for Australia (ERA)	A framework used in Australia to measure the performance and quality of research, currently for accountability and advocacy purposes	Assesses quality, volume, application of research (impact), and measures of esteem for all Australian universities at disciplinary level; does not capture societal or environmental impacts comprehensively	Bibliometrics, peer review	Compliance from the research community; burden on participants is moderate; Data accessible (engagement indicator driven); Produces metrics used for ranking; Recognizes multidisciplinary work	Indicator driven to capture engagement only; Use of peer review limits objectivity; Limited to Australian use; Less availability of indicators; Requires some central expertise (e.g., bibliometric expertise on panel)
Faster Cures Biomedical Ecosystem Metrics Project	Promotes a high performing, patient-centered biomedical systems and developing metrics to address efficiency and effectiveness of the processes	Process efficiency and effectiveness, productivity, and transparency	Early stage of development	Developed by diverse stakeholders	Under development; Focuses on biomedical innovations; Patient-centered
National Institute of Health Research (NIHR) Dashboard	A framework that consists of a dashboard to monitor the performance of research funded by the National Institute of Health Research in the UK, drawing on a logic model and a balanced scorecard approach. It accumulates data from a series of dashboards at lower levels of aggregations and is intended to be used for strategic decision making and analysis	Data collected quarterly at project level on inputs, processes, outputs and outcomes for financial, internal process and user satisfaction	System level dashboard; Peer review; data mining	Aligned with institutional goals; Can be used for monitoring impact; Comparable within organization; Indicator set is balanced; Strong theoretical basis; Wide applicability across the organization; Focused and selective set of indicators	At early phase of implementation; Limited to few indicators; High central burden; Reliant on information management systems; Not a comprehensive assessment
Productive Interactions (Europe)	A framework developed across several countries in Europe and for multiple disciplines. It is a flexible approach which aims to help institutions learn and improve their performance against their own goals. Measures productive interactions with stakeholders that lead to change	Intended to work in a wide range of contexts, best applied at research group or department level where goals are consistent	Interviews, document review, data mining	Tailored to assess performance improvements; Formative; Sensitive to organizational goals; Comprehensive; Flexible; Some tools and “how to” guides; Avoids time lag interactions to impact thus reducing bias against early career researchers; Multi-disciplinary; Broad scope suitable for a wide range of contexts	Does not produce comparison between institutions; High burden on participants; Challenging to implement; Requires assessors to identify productive interactions; Assumes interactions are a good indicator of impact
Research Excellence Framework (REF)	A framework developed to assess the performance of universities in the UK and to determine funding allocation, taking into account wider nonacademic impacts of research	Assessment at subject level on three elements: quality of research outputs, impact of research (not academic) and vitality of environment	Bibliometrics, peer review, survey, and case studies	Suitable for similar cross institutional assessment of performance; comprehensive (includes societal impact); multi-method and multidisciplinary; successfully piloted and implemented; produces a single performance indicator which can be used for ranking; acceptable to UK academic community	High burden and expensive; can discriminate against some researchers and institutions; summative; scalability not demonstrated; not transparent; almost solely reliant on peer review—limits objectivity
Snowball Metrics	A set of metrics for effective and long-term institutional research information measurement. Research metrics developed by research-intensive universities to set standards	Benchmarking	Peer/Expert review, Balanced Scorecard	Triangulate information from different data sources; Helps to understand institutional strengths and weaknesses; Free of charge	Covers only universities
Star Metrics	Its goal is to document the outcomes of science investments to the public by developing an open, automated data infrastructure and tools that will enable the documentation and analysis of a subset of the inputs, outputs, and outcomes resulting from the federal investments in science, largely to assess the performance of research and researchers for accountability purposes	Job creation; range of research funded researcher interactions and wider impacts	Data mining—collects jobs data; university administrative databases	Data mining approach is relatively novel; Minimizes burden and maximizes accountability; low participant burden once set up; not a ranking approach; does not produce a single indicator of comparative performance	Not fully developed; Not comprehensive; Summative (at present); not a ranking approach

*Sources: (1) Measuring research: A guide to research evaluation frameworks and tools—https://www.rand.org/pubs/monographs/MG1217.html; (2) Assessing the impact of healthcare research: A systematic review of methodological frameworks—https://journals.plos.org/plosmedicine/article?id=10.1371/journal.pmed.1002370*.

For example, the Research Excellence Framework is useful but focused on assessing the performance of universities to determine funding allocation; while the one used by the British National Institute of Health Research is complex and detailed with a high data collection burden (Table 1). The Canadian Academy of Health Science Framework provides consistency and comparability between institutions in a research system, consistent data gathering and presentation but is resource intensive, very complicated and has fewer standardized definitions. The Productive Interactions Framework, used widely in Europe, is broad, multi-disciplinary, and focused on individual institution's research goals, but does not allow for comparisons between institutions. The Snowball Metrics was actually developed by research-intensive universities for the primary goal of institutional benchmarking (and not internal assessments); it also utilizes fewer data sources and does not cover all impact metrics across needed domains. Further examples detailing the strengths and limitations of each framework we analyzed can be found in Table 1.

These frameworks indicate that utilizing a combination of qualitative and quantitative indicators often produces better result for research ROI assessments (Table 1). Moreover, a single metric may not tell the whole story about the outcome or impact of research, hence developing a practice of employing multiple metrics is beneficial. Each metric should be regularly evaluated for relevance to ensure alignment with organizational goals and changing research ecosystems and processes. While adopting a comprehensive approach to include all relevant metrics in an ecosystem is useful, it is also important to manage the data collection and reporting burden on the administrative staff and research community. It is also crucial to establish credible, acceptable, and customizable research metrics for an institution that take into account the views of stakeholders and interest groups. These lessons have informed the development of the proposed research measurement framework below.

## Proposed Research Metrics Conceptual Framework

### Overview

**Figure 1:**
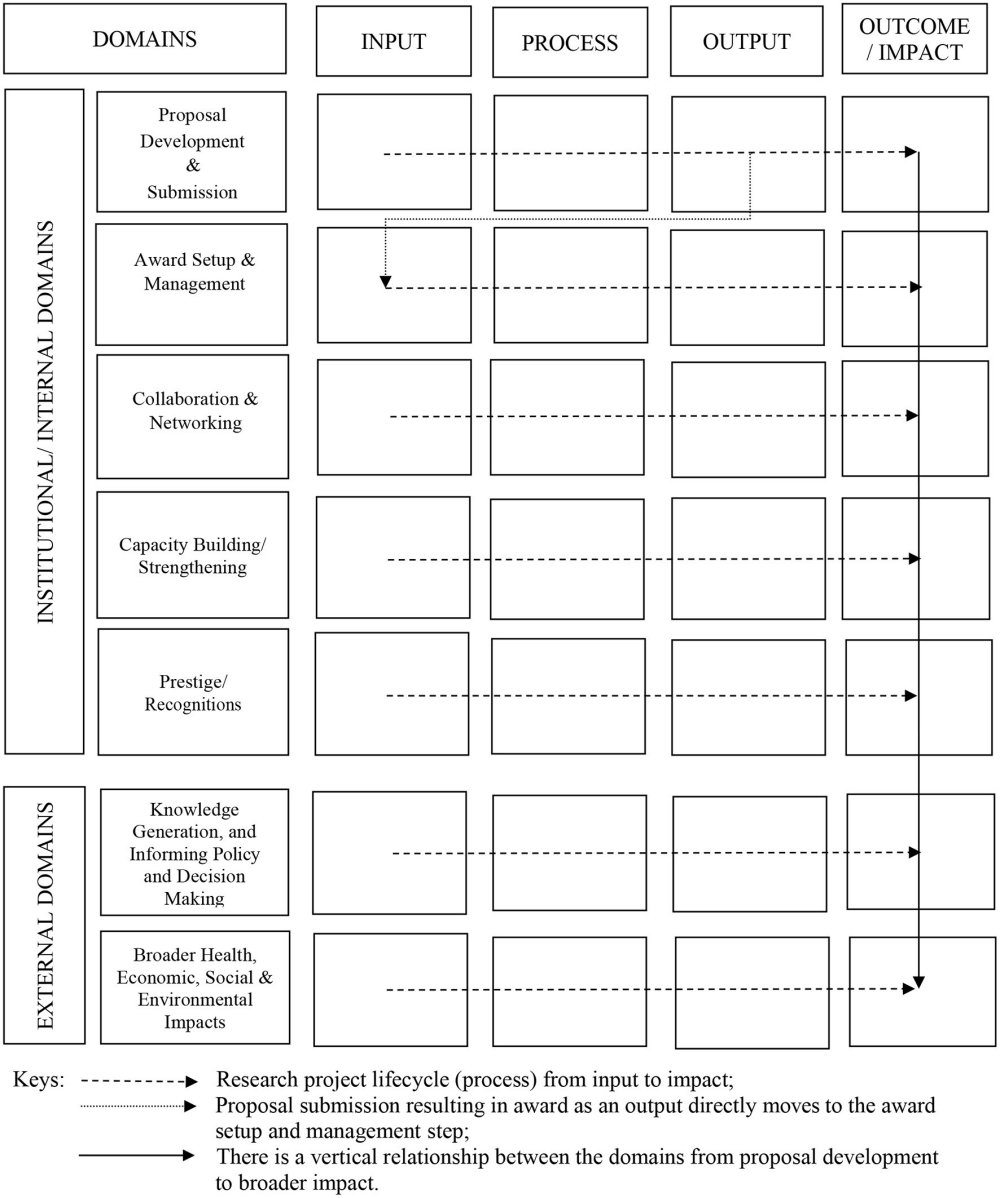
Proposed research metrics conceptual framework.

Our proposed research metrics framework uses a systems approach as one axes of measurement: Input, Process, Output, and Outcome/impact (Figure 1). On the other axes are seven domains that encompass the research lifecycle from proposal development to outcomes in society; and offer both an intra- and extra-institutional components. A portfolio of institutional research performance metrics corresponding to each domain and system category can be developed within each cell (Table 2). Each indicator can be selected to have some desired characteristics of a realistic metric, such as validity, credibility, responsiveness, reliability and availability (David and Joseph, 2014).

**Table 2:** Example research metrics for a school of health science (e.g., medicine, public health, and biomedical Science).

**Metrics**	**Organizational goal(s) alignment**	**Data source**	**Parameters/data elements/fields**
Research proposal application success or “hit” rate	Increasing the ratio of awards to proposal submissions	Internal Database	Number of proposal submitted, Number of proposal awarded, Fiscal Year (FI) of Award Date, and Department
Research Proposal Development Time (RPDT) from research proposal initiation (expression of “intent to submit”) to proposal submission to the sponsor	Increasing the administrative support to researchers to enable faculty to carry out larger and more complex research efforts, including international research	Internal Database	PI, FY (Submission Date), Project Start Date, Department, Funding Agency, and Title Category (research area)
Number and dollar amount of research grants (awards) (received)	Increasing the ratio of awards to proposal submissions; Encouraging average individual faculty and staff research productivity as a whole, as measured by: extramural direct research support, FandA support, and average proportion of faculty time devoted to research	Internal Database	PI, FY, Department, Sponsor, Title Category, Award Date, Total Dollar Amount of Funding, Type of Application (New, Continuation, Renewal, Resubmission), Sponsor Type (Federal, State, etc.), and Foreign National Involved (Yes/No)
Number and dollar amount of research projects engaging community partners	Building partnerships as measured by research in collaboration with other disciplines and/or academic institutions; community engagement; and broadening support by government agencies, foundations, industry and other funders; Increasing the capacity to carry out research in partnership with communities both within the DC area and globally	Analysis of Research Administrative Documents, PI Survey	PI, Project Start and End Date
Number of outlets for research funding opportunities announcement including availability of automatic notification and their respective views (infrastructure)	Fostering the integration of methodologic expertise, to support university research	Internal Database	Outlet Name (type), Outlet Description, Count of Views, and Automatic Notification Available (Y/N)
Number and type of honors/prizes received	Recognition of faculty, staff and students by pre-eminent science and professional societies and other bodies	Internal Database, PI Survey	Faculty/PI Name, Honor/Prize Type, Honor/Prize Date, and Honor/Prize By
Number of citations	Measuring the scientific impact of research by numbers of peer reviewed publications; impact factors for peer reviewed journals; contribution to significant scientific, health administration and policy innovations nationally and globally; and citations in scientific journals	Funders/Donors, Bibliometric Analysis, PI Survey, Review of Key Policy Documents	Publication Type (article, book, etc.), Author, Title (research area), Institution, Collaborators (if any), Publication Date, Citation Date, Cited In (Policy Documents, Clinical Guidelines, etc.), etc.
Amount of direct employment and local spending	Increasing the return on investment from research support strategies: faculty start-ups, and protected research time; pilot funds; staff training; cost-sharing; and investments	Labor Market Analysis, STAR Metrics Data, Expenditure accounts (local spending data)	Employee ID, Position Title (Role), Local/Outside, $Amount (income, etc.), Item Name, and $Amount of spending

### Axis 1: Research Lifecycle Categories

Metrics for each of the four system stages were identified through a research logic model of: (1) idea inception, (2) funding, to (3) wider benefit as follows (examples in Table 3).

**Table 3:** Research metrics portfolio (sample excerpt).

**Metrics**	**Type of Measure**	**Domain**	**Purpose/ Description**
Research proposal application success or “hit” rate	Output	Proposal Development and Submission	This metric captures the proportion of grant applications submitted to the sponsors that are successfully resulted in granting awards
Research Proposal Development Time (RPDT) from research proposal initiation (expression of “intent to submit”) to proposal submission to the sponsor	Process	Proposal Development and Submission	This metric aims to measure the time from Principal Investigators (PIs) intent to submit a research proposal to proposal submission date (define research start date)
Number and dollar amount of research grants (awards) (received)	Output	Award Setup and Management	The number and total dollar amount of all research funding awards made to school
Number and dollar amount of research projects engaging community partners	Impact	Collaboration and Networking	This metric captures absolute number and/or proportion of research projects that engage community organizations
Number of outlets for research funding opportunities announcement including availability of automatic notification and their respective views (infrastructure)	Input	Capacity Building/Strengthening	Number of outlets for research funding opportunities announcement including availability of automatic notification and their respective views
Number and type of honors/prizes received	Impact	Prestige/Recognition	The number and type of prizes and professional recognitions received
Number of citations	Impact	Knowledge Generation, Innovation and Informing Policy and Decision-Making	Number of citations of publications (on articles, policy documents, public health guidelines, books, conference proceedings, etc.)
Amount of direct employment and local spending	Impact	Broader Health, Economic, Social, and Environmental Impacts	By creating employment for both researchers and others, research activities can help reduce unemployment. In turn, newly created and filled jobs stimulate the local economy through the spending of those who fill the jobs. Local spending includes money spent on local services such as technical support, catering, and products

#### Input Metrics

Input metrics include human capital (faculty, staff, and students), finance (funding), time, infrastructure, facilities and partnerships necessary to execute research activities and produce outputs. The underlying assumption is that creating a well-equipped research environment would result in better outputs and outcomes. The optimal research input metrics demonstrate quantitative and qualitative institutional capacity measures to conduct research and produce desired outputs. It is important for an organization to identify and measure inputs to inform research activities and set organizational targets. However, there are challenges in quantifying the level of human, financial and material resources needed for investment to bring about changes within an institution and wider society. The number, composition and experience of research teams (including faculty, staff, and students) and budget line items allocated for technical and administrative costs are examples of input metrics.

#### Process Metrics

Process metrics are used to track and evaluate research performance through all phases of a project lifecycle. They serve as a measure of organizational excellence in attaining the outputs produced by way of applying specific resource inputs together. Defining and implementing appropriate research processes creates a strong bridge to effectively transform research inputs into desired outputs. Process metrics may include efficiency, effectiveness, capacity, productivity, benchmarking, and research development time based measures.

#### Outputs

Outputs are products directed to beneficiaries or stakeholders that ultimately either are of valuable milestones or bring about desired changes. Outputs can be measured in a wide variety of ways such as the number of publications in peer-reviewed journals, number of patents acquired, and amount of research expenditures. They are generally measured by the volume and quality of immediate research products that a researcher, department, or institution, produces within a specified time frame.

#### Outcomes or Impacts

Outcomes or impacts refers to demonstrable contributions that research makes at the societal level to the economy, culture, public policy, services, health, environment, or quality of life, beyond simply adding to academia. Some metrics such as number of citations, downloads, and mentions in social media can be measured in the short-term; while others such as start-ups, revenue from commercialization, broader health, and economic impacts of research are captured through long-term tracking.

### Axis 2: Research Lifecycle Domains

Each metric has been classified into a particular type of domain where all metrics share common characteristics that also facilitate data collection, analysis, and reporting (Figure 1). The domains are also broadly divided into five institutional/internal and two external/impact ones to represent the most important metrics that measure institutional performances vs. larger impacts for public health. Each domain is further descried below.

#### Proposal Development and Submission

Metrics in this category measure research inputs, processes, and outputs from proposal development, as well as individual and institutional metrics resulting from relevant processes. Examples include the number of eligible faculty participating in research development, time interval from expression of interest/funding opportunity to submission by the principal investigator (PI) to the funding agency (sponsor), and number of proposals developed and successfully submitted to funding agencies. These are generally internal/institutional pre-award performance metrics.

#### Award Setup and Management

Following receipt of grant funds after a submitted proposal is awarded, all the post-award research activities to the point of award closeout are captured within this domain. Examples include the number and aggregate dollar amount of awards received and the number of active projects. These are generally internal/institutional post-award performance metrics.

#### Collaboration and Networking

These metrics assess collaboration and networking with internal and external partners. They encompass diverse approaches to improving the culture of engagement with individuals, domestic and international organizations, communities, industry and other research partners. The metrics can measure the strength of inter- and multi-disciplinary research and the degree to which the research engages other stakeholders. Connections with various parties can help manage successful research outcomes and may include work with funders, governments, academic institutions, and industry partners. Higher numbers of interactions among various parties can be indicative of impactful production of research outputs. Collaborations (national or international) and networks can be established at the institutional level or by an individual researcher affiliated with the institution.

#### Capacity Building/Strengthening

This is a process of maximizing human, financial, material, and other resources to carryout activities effectively to consistently produce better results in achieving visions and goals of an organization. These metrics may include research infrastructure development, trainings, fellowship, participation of post-doctoral and graduate students (Masters and PhD), and academic career advancements. Capacity building is integral to any research activity, from the individual researcher, to management, and leadership staff. Examples of key infrastructure include the availability of automated systems for applications, systems for funding opportunity notifications and the number of research faculty and staff that are hired post-training.

#### Prestige/Recognition

These are measures of reputation attained by researchers (e.g., faculty, staff, students, and alumni) because of quality research efforts, such as awards and professional recognition.

### External Impact Domains

#### Knowledge Generation, Innovation, and Informing Policy and Decision-Making

The generation and use of knowledge can have a significant impact on communities, organizations, and individuals. The impact of research also extends to regulators, legislators and policymakers as it helps them develop guidelines, resolve public health issues, and adjust future strategic plans accordingly. Example of indicators in this domain include number of article and patent citations, number of editorships in high-profile journals, and number of appoints to policy groups.

#### Broader Health, Economic, Social, and Environmental Impacts

These metrics include the wider and longer-term benefits to society, depending on the type of research conducted. Such benefits include innovations, practices, services, and many other holistic improvements attributed to a number of contributors. These metrics are usually difficult to quantify and attribute to a particular organization or entity but include things like declines in disease prevalence; improvements in quality of care and service delivery; reduced unemployment; benefits from commercialization of research products; and advances in community awareness in healthcare utilization.

### Standardizing Research Metrics

The use of standard terminology and definitions is integral to research performance and impact measurement systems. Defined terms provide clarity and consistency in communicating outcomes and helps improve the standardization process. In research metrics, there has been a lack of shared conceptual clarity and inconsistent use of definitions and terms, which has in turn created confusion, duplication, and inefficiencies for research stakeholders (Remme et al., 2010). Developing uniformity in the definition of terms, data and metrics is also critical for conducting balanced comparisons among peer institutions.

Each metric draws on data collected through one or more data sources that can be used individually or in combination; and the proposed framework allows all appropriate methods of data collection, aggregation, and analysis across research proposals, awards, projects, publications, and institutions. Table 4 presents examples of most common data sources and research data extraction methods across institutional and external impact domains; for example, research publications as outputs are often extracted from bibliometric analysis. Integration and interoperability of systems within an institution may be critical for reliably tracking and measuring research data across various systems and data sources.

**Table 4:** Examples of research measurement approaches.

**Data Source/Tool**	**Description and Benefits**
Balanced Scorecard	Mostly used for quantitative performance measurement. Provides the capability to maintain big-picture long-term organizational success by integrating performances across domains and research lifecycles. It also helps to align research metrics with strategic objectives
Bibliometric Analysis	A range of techniques for assessing quantity, dissemination and content of publications and patents uses quantitative analysis to measure patterns of publications and citations. Bibliometric analysis is one of the important tools and processes used to measure research outputs such as publications and citations. It uses one or a combination of publication and citation tracking databases such as Scopus, Web of Science, PubMed, and Google Scholar to generate measures. Understanding the various types of bibliometric measures and their limitations helps to identify the appropriate ones. Bibliometrics are most useful when employed in conjunction with other measures to assess the categorical or non-comparative research outputs and impact
Case Studies	Can be used in a variety of ways; flexible enough to capture a wide variety of impacts, including the unexpected, and can provide the full context around a piece of research, researcher, or impact
Data Mining	Allows access to and understanding of existing data sets; uses algorithms to find correlations and patterns and present them in a meaningful format, reducing complexity without losing information
Institutional databases and systems	Standalone or integrated Internal database systems or applications for tracking, collecting, analyzing, and reporting research data
Interviews	Used to obtain supplemental information on areas of interest, generally to access personal perspectives on a topic, or more detailed contextual information. The participants may include PIs, staff, students, alumni, etc.
Labor Market/Economic Analysis	Provides labor and economic data to measure socio-economic returns of research
Peer Review	Review by peers, typically other academics in the same or a similar field, of outputs of research; rationale that subject experts are uniquely qualified to assess the quality of the work of others
Review of documents	Review of existing internal/external administrative or technical documents, guidelines, reports, or archives
Surveys	Provide a broad overview of the current status of a particular program or body of research; widely used in research evaluation to provide comparable data across a range of researchers and/or grants which are easy to analyze. The participants may include PI, staff, alumni, etc.

Every research activity or process has some data associated with it; but not all of this data is measured or recorded even though it may be necessary for relevant research metrics. Research data points and fields needed in each research lifecycle domain should be identified and clearly defined prior to the selection of relevant metrics. The unit of analysis and reporting is largely determined by the intended use of data; for example, some rates may be reported per PI, while others per proposal or per grant. These are some of the key decisions that institutional leadership will need to take prior to implementation of the proposed framework.

## Discussion

An internal assessment of an institutional research ecosystem can begin with discussions amongst leadership, faculty, staff, and research administrators on defining the need for research metrics and its implementation prioritized according to resource availability. Research metrics ought to align with an organization's strategic goals. The proposed conceptual framework above serves as a foundational roadmap that will help initiate discussions among an institutions' research community regarding metrics and measurement systems that fulfill local needs. The collection of standardized metrics will enhance uniformity and reliability; and improve research tracking and measurement systems across academic institutions. This framework hopes to initiate such dialogue and serve as a potential tool to better understand research and guide investment decisions for the research community and stakeholders.

Implementing a research metrics framework contributes toward quantifying the ROI on research both internally within an institution and externally in society. Illustrating the value of research is crucial in sustaining donor and taxpayer support, informing policies, and highlighting broader societal benefits of research (Guthrie et al., 2017). The use of value measures, with tangible and intangible benefits, can support advocacy for investments and give decision-makers the opportunity to draw on evidence to inform advocacy and prioritization of action (Hunter et al., 2018). If implemented successfully, a research metrics framework can also help evaluate the performance of a research system against baseline measures, and provide feedback to guide evidenced-based practice.

There are factors that challenge the systematic tracking and measurement of research performance and impact in academic institutions. For instance, lack of common understanding and agreement among groups of diverse stakeholders (with varying interests on research metrics and impact assessment) can create problems in measurement. Additionally, the absence of a universally accepted standard research measurement system and set of metrics that appropriately assess outputs and impacts makes it more difficult (Boaz and Hanney, 2020; Hanney et al., 2020). Further, choosing a comprehensive approach may create a burden on data collection and tracking. Even with the selected relevant metrics, it is not uncommon to face difficulties in ensuring data availability and quality within institutions.

Limitations in human, financial and material resources and infrastructure is a common factor hindering the generation, development, integration and automation of ideal research performance and impact measurement systems (Boaz and Hanney, 2020). As is true for other research measurement systems, impact measurement is a challenge because it is hard to attribute (or accurately quantify) the contribution of an institution's research performance to a specific group or factors. For some research, relatively longer periods of time may be necessary to observe and produce impact, hence, making it challenging to track and measure (Bornmann, 2017). The most popular bibliometric databases such as Web of Science, Scopus, PubMed, and Google Scholar do not capture the entirety of research outputs and impacts. Benchmarking and ranking metrics also require peer institutions to have similar metrics for sensible comparisons.

Integrating systems is vital to track and measure relevant metrics in each part of the research lifecycle and minimizes the reliance on one measure. This framework proposes a comprehensive approach to interrelate the input, process, output and outcome/impact metrics to have a broader picture of research works. The framework tries to triangulate information from various data sources not relying on a few measures but a combination of metrics across the domains and research lifecycle.

The benefit of this conceptual framework is that it tries to adopt the strengths of existing metrics and address the gaps observed for current frameworks (as assessed in Table 1). It recommends integration of appropriate data sources and accommodates relevant comprehensive research metrics that can be applied across institutions with less burden. The purpose of this framework is to have comprehensive research metrics that can be used across health science institutions with less cost, complexity, data collection burden, inconsistency of definitions, and issues of scale as compared to existing frameworks. Moreover, the use of a research lifecycle approach to classifying research metrics employed by this framework provides a distinctive approach to measuring ROI.

This framework also proposes a *systematic* approach to include relevant research metrics and connect input, process, output, and impact for efficient tracking and measuring of research. The most popular frameworks reviewed in this paper have several limitations in scope, scale, standards, inclusion of relevant metrics, and laying out clear processes of research lifecycle. The practical implementation of the proposed framework within and across organizations can be realized overtime to help with key decisions including allocation of resources. This paper helps conceptualize and establish a broader understanding of such systematic research tracking and measurement at different levels with more efficiency to quantify ROI of research investments across the research lifecycle—to our knowledge for the first time.

Finally, this framework incorporates approaches such as comprehensiveness, standardization, integration, research lifecycle classification, and consistency in concepts to help in the advancement of research measurement especially now when the field is still evolving and there is no single approach that is universally accepted. To reduce the burden of implementation and to enhance successful operationalization, a gradual scale up and close collaboration with research and administrative leadership within institutions and externally with peer institutions are recommended as this is tested over time.

This proposal is at the conceptual stage that needs to be tested at various levels and replicated across various institutions. The proposed framework can be piloted within the context of a particular institution from small to large scale; and the availability of resources can determine the use of such an integrated research systems to optimize effective testing and implementation. The research community can further internalize this concept, collaborate and test this framework within specific settings in the future and generate sufficient data for better application.

Overall, stakeholder interest and demand for the evidence-based evaluation of research is growing locally and internationally; most appear to be committed to better understanding and measuring research output and impact (Boaz and Hanney, 2020). Advancements in technology and data mining techniques have made tracking, extraction, analysis and reporting (as well as data availability, accessibility, and quality), more efficient and effective. Research-intensive universities and those aspiring to become one ought to promote standard tracking and measurement of research output and outcomes. Although evolving gradually, efforts are being made by a number of governmental and non-governmental institutions to develop and institutionalize frameworks that systematically measure research productivity and impact (Hanney et al., 2020). We join that movement and hope our proposed framework will stimulate a global dialogue on the value and consistency of such measures across health science schools and universities.

## Data Availability Statement

The original contributions presented in the study are included in the article/supplementary material, further inquiries can be directed to the corresponding author/s.

## Author Contributions

All authors listed have made a substantial, direct, and intellectual contribution to the work and approved it for publication.

## Funding

This research was supported by the Office of Research Excellence, Milken Institute School of Public Health, George Washington University.

## Conflict of Interest

The authors declare that the research was conducted in the absence of any commercial or financial relationships that could be construed as a potential conflict of interest.

## Publisher's Note

All claims expressed in this article are solely those of the authors and do not necessarily represent those of their affiliated organizations, or those of the publisher, the editors and the reviewers. Any product that may be evaluated in this article, or claim that may be made by its manufacturer, is not guaranteed or endorsed by the publisher.
